# Emerging insights into transcriptional condensates

**DOI:** 10.1038/s12276-024-01228-9

**Published:** 2024-04-25

**Authors:** Kwangmin Ryu, Gunhee Park, Won-Ki Cho

**Affiliations:** 1 Department of Biological Sciences, Korea Advanced Institute of Science and Technology (KAIST), 291 Deahak-ro, Yuseong-gu, Daejeon, 34141 Korea; 2 KAIST Stem Cell Research Center, Korea Advanced Institute of Science and Technology (KAIST), 291 Deahak-ro, Yuseong-gu, Daejeon, 34141 Korea

**Keywords:** Transcriptional regulatory elements, Single-molecule biophysics

## Abstract

Eukaryotic transcription, a fundamental process that governs cell-specific gene expression, has long been the subject of extensive investigations in the fields of molecular biology, biochemistry, and structural biology. Recent advances in microscopy techniques have led to a fascinating concept known as “transcriptional condensates.” These dynamic assemblies are the result of a phenomenon called liquid‒liquid phase separation, which is driven by multivalent interactions between the constituent proteins in cells. The essential proteins associated with transcription are concentrated in transcriptional condensates. Recent studies have shed light on the temporal dynamics of transcriptional condensates and their potential role in enhancing the efficiency of transcription. In this article, we explore the properties of transcriptional condensates, investigate how they evolve over time, and evaluate the significant impact they have on the process of transcription. Furthermore, we highlight innovative techniques that allow us to manipulate these condensates, thus demonstrating their responsiveness to cellular signals and their connection to transcriptional bursting. As our understanding of transcriptional condensates continues to grow, they are poised to revolutionize our understanding of eukaryotic gene regulation.

## Dynamic transcriptional condensates: enhancing eukaryotic gene expression efficiency

This review delves into the intricate realm of transcriptional regulation, a process that shapes the unique gene expression profiles of different cell types. It focuses on recent advances in elucidating the composition, property and role of transcriptional condensates, which are recently identified liquid-like entities composed of various proteins involved in transcription. Transcriptional condensate is the embodiment of ‘transcription factory’ concept, which contains high concentration of transcriptional proteins and exert control over neighboring genes. Using advanced imaging techniques, researchers found that these condensates dynamically interact with gene promoters to regulate transcription. They can also assemble in response to cellular signals, thus optimizing transcriptional efficiency and specificity. The review concludes that the dynamic relationship between these condensates and gene expression patterns is a promising area for future research and medical advancements.

This summary was initially drafted using artificial intelligence, then revised and fact-checked by the author.

## Introduction

Transcriptional regulation serves as a foundational cellular process that shapes the expression profiles of cell type-specific genes. Despite the presence of approximately 20,000 coding genes in the human genome, individual cell types selectively express only a subset of these genes. Transcription arises from intricate biochemical reactions that are coordinated between the genome and numerous nuclear proteins that are responsible for transcriptional control. Over the course of scholarly investigations in molecular biology, biochemistry, and structural biology, we have gained profound insights into these biomolecular dynamics. Furthermore, contemporary discourse has expanded to include the roles of noncoding regulatory elements and epigenetic modulation mediated by histone modifications. However, the pursuit of empirical evidence supporting our models of transcription mechanisms within the cell nucleus continues.

The cell nucleus, which is a spherical structure with an average diameter of 10 micrometers, contains and protects 3 billion DNA base pairs, comparable to a 2-meter-long strand, along with more than 6000 diverse proteins. This subcellular organelle coordinates vital genetic processes such as DNA replication, transcription, and posttranscriptional regulation, making the nuclear environment considerably more compact than the surrounding cytoplasm. Historically, transcription within the nucleus was theorized as a stochastic interaction driven by protein‒DNA diffusion and collision. However, recent studies using fluorescently labeled nuclear proteins have revealed constrained movement patterns within the nucleus. This result suggests that simultaneous multiprotein assembly at a single gene site by diffusion is unlikely.

A groundbreaking experiment using Br-UTP-labeled nascent RNA redefined this understanding by identifying transcription hotspots in the nucleus^1^. This early observation shifted the paradigm from the stochastic transcription regulation model to the concept of “transcription factories”, where proteins responsible for transcription are concentrated, suggesting that transcriptionally active genes congregate in or around these specialized nuclear zones.

While initial observations using conventional microscopy supported the stationary transcription factory model, newer insights from advanced microscopy techniques introduced a different perspective^2,3^. These studies detected temporally dynamic clusters of many RNA polymerase II molecules and other transcription factor molecules, thereby challenging the static transcription factory model. Moreover, some experimental evidence has shown that the dynamic clusters of proteins responsible for transcription play a role in the transcriptional regulation of genes that exhibit bursting behaviors and are associated with super-enhancers^4–7^. Interestingly, subsequent studies revealed that the clusters displayed liquid-like properties in the nucleus of living cells, giving rise to the term “transcriptional condensates.” This discovery has altered our perspective, prompting a reassessment of established transcription models.

## Traditional eukaryotic transcription model

The foundational discovery showed that DNA serves as a repository of genetic information in living organisms was originally made by Hershey and Chase through their T2 bacteriophage experiment, in which they employed *Escherichia coli* (*E. coli*) as a prototypical prokaryote model^8^. Subsequently, studies in *E. coli* established the central dogma, which delineated the synthesis of proteins from DNA via the intermediary RNA. To demonstrate the transcription process within prokaryotic environments, a stochastic transcription regulation model based on protein‒DNA interactions via diffusion and collision was proposed.

Although the stochastic transcription regulation model has provided valuable insights into transcriptional dynamics, its applicability in elucidating the intricacies of eukaryotic transcription remains debatable^9^. One fundamental difference between prokaryotic and eukaryotic organisms is the presence of the nucleus, coupled with a sophisticated nucleosome architecture governed by histones. Furthermore, eukaryotic transcription is characterized by a markedly more complex assortment of transcription factors than prokaryotic transcription. Initiating transcription from a single gene locus in eukaryotic cells requires the assembly of several proteins at a specific genomic locus. Given the dense molecular environment within the nucleus of eukaryotic cells, the feasibility of such a coordinated assembly remains a subject of academic inquiry.

One particular challenge for the stochastic model is explaining transcription bursts, wherein many mRNAs are explosively synthesized from a single gene locus in a brief time frame^10,11^. External stimuli drive cell differentiation or state changes, thus prompting the activation of numerous genes in a rapid temporal sequence. For example, in response to serum treatment following serum starvation-induced G0/G1-phase cell arrest, specific genes under the control of serum response factors are activated. Notably, the β-actin gene is a prominent gene known to be involved in transcription bursting under serum stimulation.

Observations made via MS2 tagging have allowed real-time monitoring of nascent β-actin gene (*Actb*) spots in living mouse cells^12^. According to experimental findings, the activation of *Actb* transcription commences within minutes of serum addition, leading to the rapid synthesis of substantial mRNA quantities before returning to basal transcription levels. It is challenging to adopt traditional stochastic transcriptional regulation paradigms to explain this gene bursting phenomenon. Given the presence of numerous general transcription factors and the capacity of a single transcription complex to synthesize only one mRNA at a time, the following question arises: How does serum stimulation timely orchestrate the convergence of the numerous requisite proteins for transcription bursting at a single *Actb* gene locus?

The previously posited transcription factory model offers a compelling framework for elucidating transcription bursting events within the eukaryotic cell nucleus. The model predicts that the biomolecules essential for transcription are pre-assembled within a spatial domain, and the interaction between this domain and the activated gene region drives the burst of transcription. Thus, the remaining questions are whether the genes migrate to this spatial domain or whether this domain dynamically relocates to the genes.

## Properties and composition of transcriptional condensates

The transcription factory model postulates that transcription-associated proteins, including RNA polymerase II, localize to distinct nuclear compartments. Pioneering endeavors utilized fluorescence-tagged RNA polymerase II to visualize the ‘factory’. However, the high density of these molecules within the nucleus poses significant challenges for discerning discrete foci using conventional fluorescence microscopy. Breakthroughs arose with the advent of single-molecule localization-based superresolution microscopy, which revealed discernible clusters of RNA polymerase II molecules suggestive of transcription factories, with their sizes similar or below the diffraction limits of conventional fluorescence microscopy^2^.

Indeed, superresolution microscopy revealed an inhomogeneous distribution of RNA polymerase II^2^. Through photoactivation localization microscopy (PALM), distinct RNA polymerase II clusters, which were characterized by the variations in burst durations and sizes, were identified. Subsequent studies proved that essential transcription factors, including Mediator and BRD4, similarly formed clusters in proximity to RNA polymerase II^4,5^.

Meanwhile, the liquid-liquid phase separation (LLPS) phenomenon has emerged as a prominent mechanism underlying the formation of various intracellular compartments. LLPS, which was initially documented in P granules^13^, was recognized as a ubiquitous mechanism facilitating the establishment of diverse intracellular compartments, such as the nucleoli, stress granules, and nuclear speckles^7,14–16^.

**Fig. 1:**
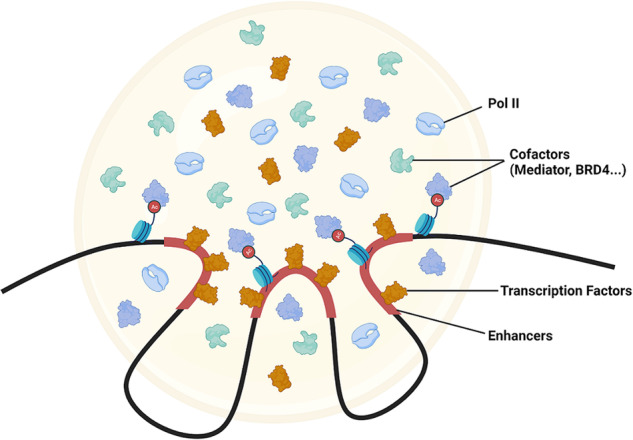
Schematic representation of the transcriptional condensate model. Transcriptional condensates are thought to form around enhancer-rich regions through liquid‒liquid phase separation.

This realization instigated a deeper exploration into the nature of the RNA polymerase II clusters detected by superresolution microscopy: Could these clusters represent phase-separated condensates? Endogenous protein labeling facilitated by CRISPR/Cas9 technology revealed that polymerase II, along with Mediator and BRD4, can form liquid droplets at physiological concentrations in living cells^4,5^. Concordant with the dynamic nature of LLPS, these droplets demonstrated rapid fluorescence recovery after photobleaching (FRAP) and fusion events upon contact within living cells, leading to their characterization as phase-separated condensates, which are referred to as “transcriptional condensates” (Fig. 1).

Time-correlated PALM (tcPALM) revealed both stable and transient RNA polymerase II and Mediator clusters within living cells^2,4^. While stable clusters comprising hundreds of Pol II and Mediator molecules correspond to conventionally characterized phase-separated liquid droplets, the exact properties of the transient clusters remain as an enigma, partly due to observational constraints. At first, ‘transcriptional condensates’ referred to stable clusters that clearly form droplets. However, the recent definition of ‘condensate’ tends to be broader—membrane-less compartments occupied by high concentrations of specific biomolecules, governed by nonstoichiometric, multivalent interactions^17,18^. Yet requiring more delicate investigation, these transient clusters might represent condensates in this ‘broader’ context, reflecting their dynamic transcriptional activity^3,19^.

Advancements in single-molecule nanoscopy, leveraging stimulated emission depletion microscopy (STED), enabled single-molecule visualization of proteins at target genomic sites^20,21^. Observations revealed clustering of ~10 RNA polymerase II, Mediator, BRD4 and transcription factor molecules at transcriptionally active genes. These clusters show a dynamic nature upon FRAP, and the numbers of constituent molecules gathered on the gene are reminiscent of the transient clusters detected by tcPALM. Although whether all the ‘transcriptional condensates’ referred to in various studies satisfy the rigorous terms of LLPS remains debatable, the tendency of transcriptional regulators to come together as dynamic condensates is apparent.

Notably, the primary components of transcriptional condensates—RNA polymerase II, Mediators, and BRD4—all possess intrinsically disordered regions (IDRs), which mediate weak multivalent interactions that are crucial for phase separation. These IDRs are essential for the integration of these proteins within condensates both in vitro and in vivo^22–24^. Moreover, specific transcription factors harboring IDRs are also found to be localized within these transcriptional condensates^25,26^. By tethering the IDRs of transcription factors to the promoter of the reporter gene, Pol II and Mediator are recruited to form condensates at the locus, and robust activation of the gene is observed. The optogenetic induction of the recruitment of transcription factors to the reporter gene also enhances transcription^27^. It has been inferred that specific transcription factors serve as nucleation ‘seeds’ for condensates to form on endogenous target loci. The ‘condensate’ concept offers a new framework for understanding how the activation domains of various transcription factors function, which often lack conserved structures.

## Transcriptional condensates formation on chromatin

Chromatin is another essential component involved in the formation of the transcriptional condensates. While the proteins composing the condensates have the intrinsic properties required for phase separation and droplet formation in vitro, the physiological concentrations of these proteins are much lower in vivo. DNA and histones on chromatin can offer multiple binding sites for these proteins to add another layer of multivalent interactions, which leads to the formation of transcriptional condensates in the nuclei of living cells.

Experimental evidences support indispensable role of chromatin in transcriptional condensate formation. Treatment of JQ1, a BET bromodomain inhibitor that blocks the interaction between BRD4 and acetylated histones, abolished Pol II and Mediator cluster formation in mouse embryonic stem cells^4^. Concurrently, acute depletion of the chromatin architectural protein CTCF similarly negated transcriptional condensate formation in HCT116 cells^28^. These findings underscore the chromatin specificity of transcriptional condensates.

Conversely, paused Pol II reinforces enhancer–promoter interactions^29^ and microphase separation of Pol II, RNA–binding proteins, RNA and chromatin is important for maintaining euchromatin organization in the ‘microemulsion’ state^30^. Therefore, the function and structure of euchromatin are inseparable and they contribute to the maintenance of one another. Multivalent interactions, which induce the local condensation of molecules, are now recognized as key determinants of euchromatin organization.

The exact site of transcriptional condensate formation within the nucleus is still unclear. Yet, super-enhancers, which are broad genomic regions marked by distinct ChIP signals for Mediator and active chromatin markers, have been identified as probable sites for the formation of transcriptional condensates^6,31^ (Fig. 1). Given the numerous binding sites that super-enhancers offer to the components of transcriptional condensates, they are considered potential epicenters for initiating condensation. The assembly of transcription-related proteins around essential genes, orchestrated by super-enhancers, might be a critical mechanism that reinforces the consistency of transcriptional identities across cells.

Given the cell-specific landscape of transcription factors and activated enhancers, one can infer that the composition and characteristics of transcriptional condensates differ across cell types and contexts. As such, a comparative analysis across diverse cellular environments may be needed to gain a better understanding of transcriptional condensates.

## Spatiotemporal interplay between transcription and transcriptional condensates

Although the precise causality between transcriptional condensates and transcription remains unclear, a plethora of evidence indicates that these condensates play a pivotal role in transcription. Live-cell imaging revealed that serum stimulation give rise to prominent clusters of Pol II at the *Actb* locus, which is a well-known target of serum stimulation. The lifetime of the cluster was proportional to the amount of *Actb* mRNA produced, indicating that Pol II clustering is closely related to transcriptional output. RNA fluorescence in situ hybridization (FISH) of super-enhancer-associated genes in mouse embryonic stem cells (mESCs) also revealed their colocalization with Mediator, Pol II, and BRD4, which are the key elements of transcriptional condensates^4,5,23^. Transcriptionally active loci often appear to be occupied by transcriptional condensates.

**Fig. 2:**
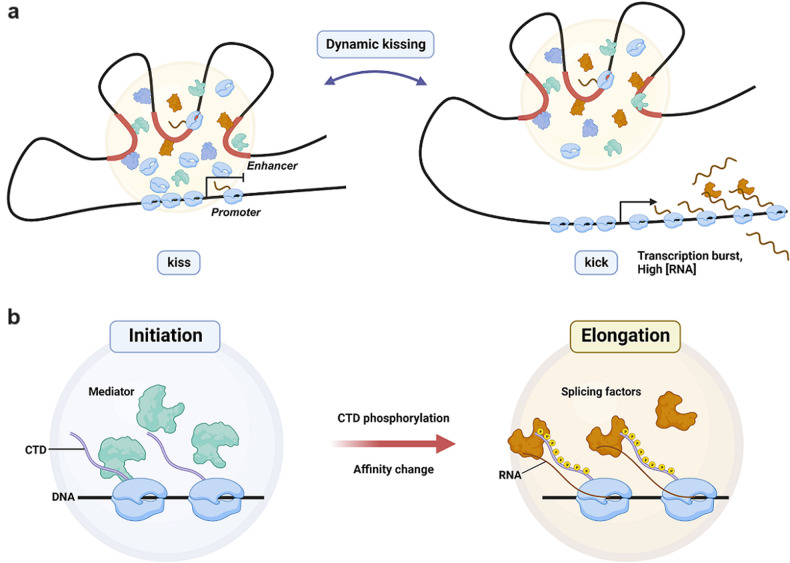
Dynamic models describing the functions of transcriptional condensates. **a** Schematic model for the dynamic interplay between transcriptional condensates and transcription. **b** Schematic model for the transition of Pol II partitioning during the process of transcription.

However, time-lapse imaging in live cells demonstrated that nascent RNA signals do not consistently overlap with transcriptional condensates. Instead, they exhibit intermittent interactions, leading to the postulation of the ‘dynamic kissing’ model^4^. This model posits that while transcriptional condensates arise at enhancer loci, they sporadically engage with promoters, thereby inducing active transcription (Fig. 2a).

More detailed experiments visualizing DNA, nascent RNA, and transcriptional regulatory factors in live cells have elucidated the spatiotemporal relationship between transcriptional condensates and transcriptional output. For the Nanog locus in mESC, Proximity of Pol II-promoter and BRD4-promoter were positively correlated with transcriptional activity, while the proximity of Mediator-promoter did not significantly correlate with the amount of RNA produced^32^. A recent report showed that deletion of the genomic region between the Klf4 enhancer and promoter reduced the Mediator-promoter distance but did not change the Pol II-promoter distance^33^. These observations imply that while Pol II and Mediator are both present in transcriptional condensates, each factor may occupy slightly different positions relative to chromatin and exert distinct effects on transcription regulation.

Consistently, the phosphorylation of Pol II, which occurs in line with transcription initiation and elongation, has been shown to reduce the affinity of Pol II for Mediator both in vitro and in vivo^23^. Phosphorylated Pol II instead gains affinity for splicing factors, which are concentrated in nuclear speckles (Fig. 2b). Notably, the produced RNA can provide feedback to transcriptional condensates. While a small amount of RNA can facilitate condensate formation, a high RNA concentration favors dissolution of the condensate; thus, explaining the underlying mechanism of transcription ‘bursts’^34^. Based on these observations, it is becoming increasingly clear that transcriptional condensates are not homogenous liquid droplets. Rather, they are complex and dynamic assemblies that change in composition during transcription. A more detailed characterization of the microstructure of transcriptional condensates may come with technical breakthroughs.

A recent innovative technique, known as chromatin expansion microscopy (ChromExM)^35^, which combines expansion microscopy and STED, achieved an ~3 nm resolution and revealed distinct types of RNA polymerase II clusters in zebrafish embryos. These include ‘macroclusters’ that were rich in RNA polymerase II and the transcription factor Nanog, similar to the larger stable clusters identified by PALM. In contrast, ‘string’ structures possibly represent elongating RNA polymerase II molecules, reminiscent of transient clusters, or clusters identified through single-molecule nanoscopy. These divergent cluster types might signify different transcriptional processes or states. Notably, treatment with the transcription inhibitor α-amanitin resulted in the disappearance of elongating ‘string’ structures, while macroclusters remained intact. Moreover, the interparticle distances between Nanog and RNA polymerase II decreased after α-amanitin treatment. These observations, considered together with the observations regarding Pol II phosphorylation, led to the proposal of the ‘kiss-and-kick’ model (Fig. 2a). Such advanced insights challenge the simplistic notion that the clustering of transcriptional regulatory factors directly equates to transcriptional activity and demonstrate a more delicate spatiotemporal interplay between transcriptional condensates and transcriptional outputs.

Due to the significant role of transcriptional condensates in transcription regulation, mutations in transcription factors in transcriptional condensates can lead to aberrations in gene expression and subsequent disease. For instance, expansions of alanine repeats in HOXD13 are associated with hereditary synpolydactyly in humans. Such repeat expansions in HOXD13 perturb its composition within transcriptional condensates, thereby altering phase separation dynamics. Consequently, segregated condensates form, leading to the dysregulation of gene expression governed by this transcription factor. Moreover, mutations in HOXA13, RUNX2, and TBP have also been shown to modulate phase separation dynamics, underscoring the broad relevance of this phenomenon in transcriptional regulation and disease pathology^36^.

## Engineering transcriptional condensates to control transcription

As evidences have emerged that transcriptional condensates may play a role in transcription regulation, researchers have begun to investigate methods for spatiotemporally manipulating condensates. Optogenetic tools have been developed to artificially increase the concentration of proteins, thus allowing for the generation of synthetic transcription condensates.

**Fig. 3:**
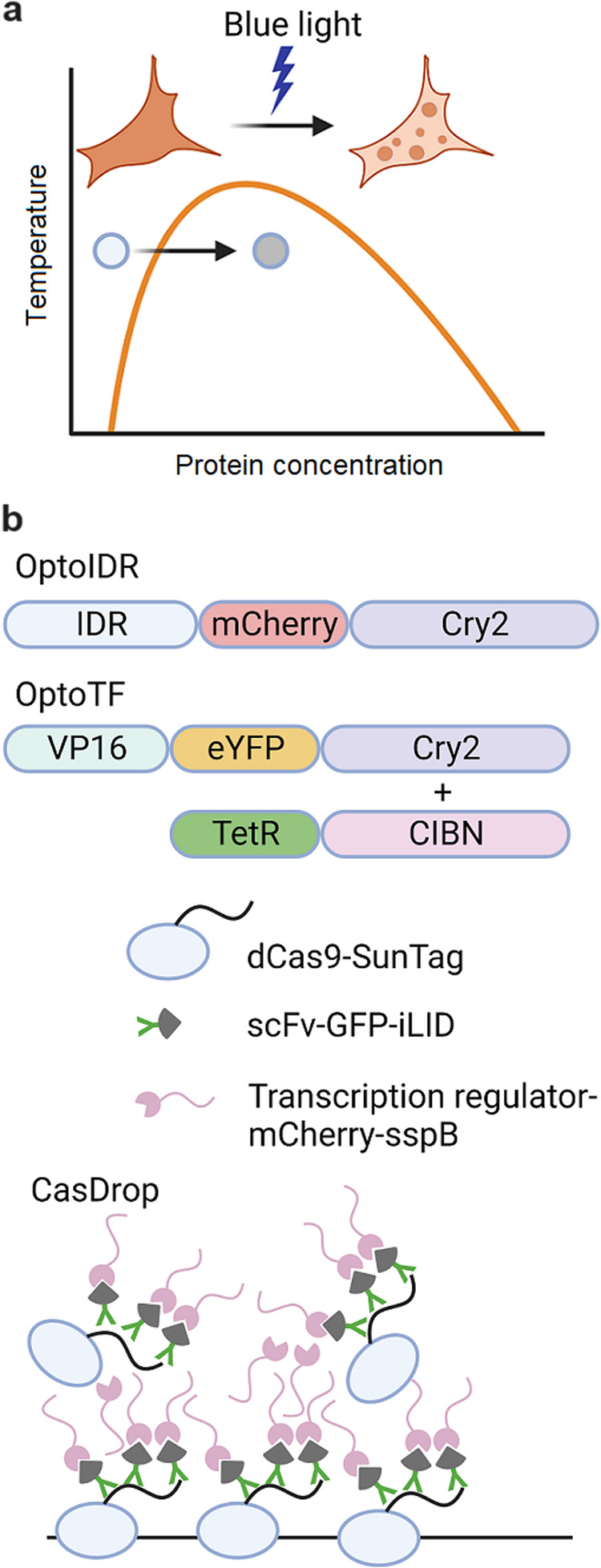
Engineering Transcriptional Condensates for Transcription Control. **a** Schematic diagram of the optogenetic control of LLPS. Blue light can activate rapid clustering of IDRs containing proteins to let their concentration surpass the critical point. **b** Various platforms of optogenetic tools for LLPS formation are shown. Each platform contains proteins that can interact with each other under blue light conditions, such as IDR, CIBN, or iLID. Transcription activator-linked optogenetic platforms are utilized for spatiotemporal control of gene regulation.

Cry2 is a plant-derived protein that undergoes dimerization upon exposure to blue light. To achieve liquid‒liquid phase separation, it is necessary to surpass a critical concentration of proteins. Therefore, a technique that utilizes Cry2 called OptoDroplet was developed^37^. In this approach, IDRs of proteins capable of LLPS were tagged with Cry2 and expressed (Fig. 3a). Upon exposure to blue light, the induction of liquid condensates was observed. In this technique, the intensity and duration of blue light exposure allowed for the control of droplet size and concentration, and localized illumination enabled the spatiotemporal induction of droplets. Subsequently, a technology called CasDrop, which utilizes dCas9 and the SunTag epitope to recruit transcription regulators to specific chromatin loci, was developed^38^ (Fig. 3b). Both of these methods leverage optogenetic control through blue light, thus enabling the creation of spatiotemporal and reversible synthetic transcription condensates.

The optogenetic toolkit capable of artificially manipulating transcriptional condensates has also been demonstrated to be applicable in the control of actual gene expression. Experimental results from the OptoTF system, where transcription factors are tagged with Cry2 and recruited to target loci, have shown successful control of reporter gene expression both in vitro and in vivo^39^. Additionally, when OptoDroplets were induced using the IDR of TAF15, they were confirmed to incorporate RNA polymerase II, and their positioning was observed at loci where transcription bursting occurs^40^.

However, contradictory results have shown that ectopic OptoDroplets of the transcription factor VPR fail to recruit Pol II or enhance transcription^27^. A study using the low-complexity domain (LCD) of the oncogenic transcription factor EWS/FLI1 concluded that only a narrow optimal interaction of LCD activates transcription, and overexpressing LCD rather inhibits EWS/FLI1 target genes^41^. Thus, when engineering transcriptional condensates, the fact that the formation of ‘functional’ condensates is highly sensitive and context-dependent must be considered. While increasing the concentration or interactions of a single factor may be sufficient to induce observable droplets, it does not guarantee the recruitment of other proteins or the normal functioning of the droplet.

## Transcriptional condensates in responses to signaling

**Fig. 4:**
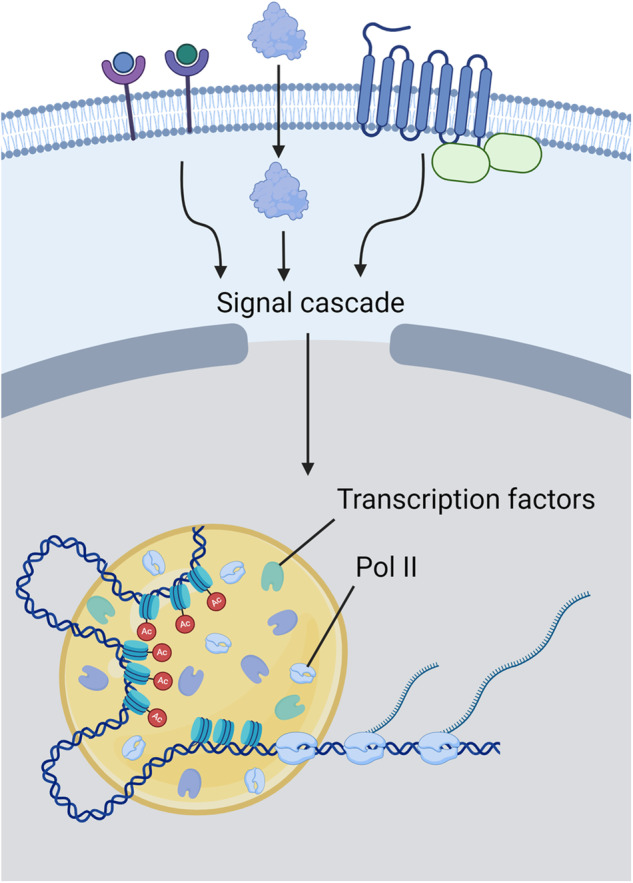
Transcriptional Condensates in the Response to Signaling. Diverse external signals are transmitted to transcriptional condensates through cell signaling cascades. Signal molecules are recruited to the target chromatin region to form LLPS droplets with transcription factors and RNA polymerase II. H3K27ac-modified chromatin (labeled “Ac”) provides a platform for recruiting various transcription factors via long-range clustering.

Cells respond to various signals or environmental changes to execute programmed responses or maintain homeostasis. Different transcription factors can be activated by different signals, and these transcription factors recruit Pol II and other general transcriptional proteins to their target genes. Therefore, transcriptional condensates may serve as the endpoint of signaling cascades (Fig. 4). Indeed, factors involved in pathways^41^ such as the WNT, JAK/STAT3, and TGF-β signaling pathways are incorporated into Mediator condensates around super-enhancers^42^. The partitioning of transcription factors around these DNA elements directly influences operation of the transcription machinery, thereby modulating gene expression.

A recent study reported that transcription factors at the endpoint of the Hippo signaling pathway, a cellular signaling pathway involved in the response to cell density or environmental stiffness, undergo phase separation to regulate gene expression. YAP has been shown to form condensates under hyperosmotic stress conditions, and these condensates colocalize with RNA polymerase II clusters within the cell nucleus. Under hyperosmotic stress conditions, YAP condensates not only regulate gene expression within the nucleus but also form cytoplasmic condensates. In this context, YAP collaborates with the Hippo pathway kinase LATS1 to form cytoplasmic condensates that differ in composition from nuclear YAP condensates, including TEAD, which is a transcriptional regulator of gene expression within the nucleus. The formation of condensates with distinct partners regulates the redistribution of YAP, suggesting that YAP controls its activity in signaling pathways by interacting with different factors in various cellular compartments^43^. Another factor in the YAP/TAZ signaling pathway, TAZ, is also known to undergo phase separation to regulate gene expression^44^. The dynamics of TAZ were observed under various environmental conditions that regulate the Hippo pathway, such as serum stimulation, cell density, and stiffness, and it was revealed that Hippo signaling negatively regulates the compartmentalization of TAZ. The TAZ condensate has been reported to include several transcription factors, including TEAD.

Androgen receptor (AR) and estrogen receptor alpha (ERα) are known as oncogenic factors that control super enhancer genes in prostate cancer and breast cancer, respectively. When these factors are stimulated, they lead to the reorganization of many gene expression programs, thereby contributing to changes in cancer progression. In this process, both AR and ERα reportedly form liquid condensates within cancer cells. In breast cancer, ligand-bound ERα genes interact with each other, forming clusters. ERα condensates induce changes in genome organization and influence the expression of cancer genes^45^. In prostate cancer, when AR is stimulated, it is recruited to super enhancers, where it forms liquid condensates. These condensates interact with MED1 and RNA polymerase II and play a role in promoting the transcription of genes involved in cancer progression^46^.

Transcriptional condensates have been shown to play a role in gene expression even under physical stress conditions such as heat shock. Cells respond to thermal stress through the expression of heat shock response genes such as those encoding chaperones. Nuclear stress bodies (nSBs) are prominent nuclear subcompartments that are formed during heat stress. Composed of proteins such as HSF1, nSB is known to form condensates with various transcription factors. These nSBs bind to chromatin containing HSP genes to promote gene expression^47^. In yeast, heat shock response condensates regulate genome organization^48^. Hsf1, along with Mediator and RNA polymerase II, forms condensates where the chromatin interaction of heat shock response genes is reorganized.

## Conclusion

Studies on transcriptional regulation have evolved profoundly over the past few decades. Traditional models, such as the stochastic and transcription factory models, have been complemented and, in some cases, supplanted by emerging insights into transcriptional condensates. These liquid-like entities formed through liquid‒liquid phase separation act as dynamic hubs that focus the transcription machinery, thereby optimizing the efficiency and specificity of transcription.

Advanced imaging techniques and molecular biology research have enriched our understanding of these condensates, revealing their transient interactions with gene promoters and adaptability in response to cellular signals. The centrality of these condensates in gene expression upon cellular signaling, combined with their potential role in pathological conditions such as cancer, underscores their significance in cellular biology.

However, a comprehensive understanding of the entire biomolecular composition of transcriptional condensates remains elusive. It is unclear which domains of the constituent molecules play a pivotal role in condensate formation and dissolution. Furthermore, the composition of transcriptional condensates may vary depending on the genes studied. A critical challenge to address is the need for robust evidence regarding how the manipulation of transcriptional condensate dynamics impacts the regulation of specific gene expression.

Finally, as we harness innovative technologies to manipulate transcriptional condensates, the prospects for therapeutic interventions expand. The dynamic interplay between these condensates and gene expression patterns presents a frontier of exploration that holds promise for future research and medical advancements.
